# Antimicrobial activity of ceftibuten-avibactam against a global collection of Enterobacterales from patients with urinary tract infections (2021)

**DOI:** 10.1007/s10096-023-04562-4

**Published:** 2023-02-22

**Authors:** Helio S. Sader, Cecilia G. Carvalhaes, Michael D. Huband, Rodrigo E. Mendes, Mariana Castanheira

**Affiliations:** JMI Laboratories, 345 Beaver Kreek Centre, Suite A, North Liberty, IA 52317 USA

**Keywords:** Urinary tract infection, Carbapenem-resistant Enterobacterales, Ceftibuten-avibactam, Extended-spectrum β-lactamase, CRE, ESBL

## Abstract

We evaluated the in vitro activity of ceftibuten-avibactam against Enterobacterales causing urinary tract infection (UTI). A total of 3216 isolates (1/patient) were consecutively collected from patients with UTI in 72 hospitals from 25 countries in 2021 then susceptibility tested by CLSI broth microdilution. Ceftibuten-susceptible breakpoints currently published by EUCAST (≤ 1 mg/L) and CLSI (≤ 8 mg/L) were applied to ceftibuten-avibactam for comparison. The most active agents were ceftibuten-avibactam (98.4%/99.6% inhibited at ≤ 1/ ≤ 8 mg/L), ceftazidime-avibactam (99.6% susceptible [S]), amikacin (99.1%S), and meropenem (98.2%S). Ceftibuten-avibactam (MIC_50/90_, 0.03/0.06 mg/L) was fourfold more potent than ceftazidime-avibactam (MIC_50/90_, 0.12/0.25 mg/L) based on MIC_50/90_ values. The most active oral agents were ceftibuten (89.3%S; 79.5% inhibited at ≤ 1 mg/L), levofloxacin (75.4%S), and trimethoprim-sulfamethoxazole (TMP-SMX; 73.4%S). Ceftibuten-avibactam inhibited 97.6% of isolates with an extended-spectrum β-lactamase phenotype, 92.1% of multidrug-resistant isolates, and 73.7% of carbapenem-resistant Enterobacterales (CRE) at ≤ 1 mg/L. The second most active oral agent against CRE was TMP-SMX (24.6%S). Ceftazidime-avibactam was active against 77.2% of CRE isolates. In conclusion, ceftibuten-avibactam was highly active against a large collection of contemporary Enterobacterales isolated from patients with UTI and exhibited a similar spectrum to ceftazidime-avibactam. Ceftibuten-avibactam may represent a valuable option for oral treatment of UTI caused by multidrug-resistant Enterobacterales.

**Supplementary Information:**

The online version contains supplementary material available at 10.1007/s10096-023-04562-4.

## Introduction

Urinary tract infections (UTIs) are among the most common of all bacterial infections. Approximately 50% of all women experience at least 1 UTI by the age of 35, and approximately 20% of women between the ages of 18 and 24 have a UTI annually [1]. The majority of UTIs are treated on an outpatient basis. However, resistance to first-line oral antimicrobials that are used to treat UTIs has increased markedly in the last 2 decades, complicating outpatient treatment approaches [2]. In the USA, *Escherichia coli* resistant to trimethoprim-sulfamethoxazole (TMP-SMX) among adult females with UTI has exceeded 25%, and among females aged ≥ 65 years, ciprofloxacin resistance approaches 30% [3]. In parts of Europe, resistance to TMP-SMX and ciprofloxacin has also increased dramatically among *E. coli* [4]. Moreover, side effects of some first-line agents for treatment of UTI are of great concern [5, 6].

Other important mechanisms of resistance in UTI pathogens are the production of extended-spectrum β-lactamases (ESBLs) and, more recently, the production of carbapenemases. These resistance mechanisms are frequently associated with fluoroquinolone and/or aminoglycoside resistance determinants [7]. As the number of pathogens resistant to outpatient therapies has risen, the number of hospitalizations for UTIs has also grown [2].

Other oral agents used to treat UTI include fosfomycin and pivmecillinam, the oral version of mecillinam. Most clinical data available is on the effectiveness of fosfomycin to treatment and prevention of lower UTI, primarily cystitis [8]. Although fosfomycin remains very active against *E. coli*, including MDR and carbapenem-resistant isolates, it has more limited activity against *Klebsiella* spp. and other Enterobacterales responsible for UTI [9]. Mecillinam is also an old antibiotic that remains very active against *E. coil* even in regions where it is commonly used to treat UTI; however, a randomized clinical trial comparing this drug with standard of care regimes is warranted [10, 11].

Ceftibuten is an oral third-generation cephalosporin which is highly potent against Enterobacterales and stable against many class A and C β-lactamases produced by these organisms, including some ESBLs [12, 13]. Avibactam is a synthetic diazabicyclooctane (DBO) non-β-lactam inhibitor. Avibactam is available for clinical use in combination with ceftazidime as an IV formulation; an avibactam formulation for oral use is currently being developed to be combined with ceftibuten for clinical use. Compared with clavulanic acid, sulbactam, and tazobactam, avibactam provides excellent inhibition of most clinically relevant class A and class C β-lactamases, such as ESBLs, KPCs, and AmpC β-lactamases [14]. In the present study, we evaluated the in vitro activity of ceftibuten-avibactam (fixed 4 mg/L) against a contemporary collection of Enterobacterales collected from patients with UTIs worldwide.

## Materials and methods

Participant medical centers were invited to collect a specific number (25 to 60, depending on geographic region) of consecutive isolates (1/patient) from patients with UTI in 2021. Only bacterial isolates determined to be significant by local criteria as the reported probable cause of infection were included in this investigation. The organism collection included 3216 isolates from 72 medical centers in 25 countries. Isolates were mainly from the US (*n* = 1585; 29 centers) and Europe (*n* = 1410; 33 centers in 18 countries), but also included *E. coli* isolates from Latin America (*n* = 121; 6 centers in 5 countries) and Japan (*n* = 100; 4 centers).

Antimicrobial susceptibility was evaluated by reference broth microdilution method in a monitoring laboratory (JMI Laboratories, North Liberty, Iowa, USA) and conducted according to Clinical and Laboratory Standards Institute (CLSI) procedures (document M07) [15]. Current ceftibuten breakpoints published by CLSI (≤ 8 mg/L) and EUCAST (≤ 1 mg/L) were applied to ceftibuten-avibactam for comparison [16, 17]. Avibactam was present at a fixed concentration of 4 mg/L in combination with ceftibuten.

*E. coli*, *K. pneumoniae*, and *P. mirabilis* isolates were categorized as exhibiting an ESBL phenotype based on CLSI criteria; i.e., the isolate had an elevated MIC value (≥ 2 mg/L) for ceftazidime, ceftriaxone, or aztreonam [16]. Isolates were considered multidrug resistant (MDR) according to criteria defined in 2012 by the joint European and US Centers for Disease Control, which defines MDR as nonsusceptible to ≥ 1 agent in ≥ 3 antimicrobial classes [18]. The following representative agents from each antimicrobial class and their CLSI interpretive criteria were: ceftazidime (≥ 8 mg/L), ceftriaxone (≥ 2 mg/L), cefepime (≥ 4 mg/L), meropenem (≥ 2 mg/L), imipenem (≥ 2 mg/L), piperacillin/tazobactam (≥ 16/4 mg/L), levofloxacin (≥ 1 mg/L), ciprofloxacin (≥ 0.5 mg/L), gentamicin (≥ 8 mg/L), amikacin (≥ 32 mg/L), tobramycin (≥ 8 mg/L), colistin (≥ 4 mg/L; resistant), and tigecycline (≥ 4 mg/L; US-FDA interpretive criteria). CRE were defined as Enterobacterales that displayed imipenem or meropenem MIC values at ≥ 4 mg/L. Imipenem MIC results were not applied to *Proteus mirabilis* or indole-positive Proteeae due to their intrinsically elevated MIC values.

## Results

The frequencies of Enterobacterales isolated from patients with UTIs in the USA and Europe are presented in supplemental Figure S1. *E. coli*, *Klebsiella pneumoniae*, and *Proteus mirabilis* were the most common species and represented 77.3% and 81.3% of organisms from the USA and Europe, respectively. Ceftibuten-avibactam inhibited 98.4% and 99.6% of Enterobacterales isolates at ≤ 1 mg/L and ≤ 8 mg/L, respectively (MIC_50/90_, 0.03/0.06 mg/L; Tables 1 and 2). Ceftibuten-avibactam was the most active oral agent, exhibiting in vitro activity similar to the most active IV agents, such as ceftazidime-avibactam (MIC_50/90_, 0.12/0.25 mg/L; 99.6% susceptible per CLSI and EUCAST), amikacin (MIC_50/90_, 2/4 mg/L; 99.1%/97.8% susceptible per CLSI/EUCAST), and meropenem (MIC_50/90_, 0.03/0.06 mg/L; 98.2%/98.3% susceptible per CLSI/EUCAST; Table 2). Notably, ceftibuten-avibactam was fourfold more potent than ceftazidime-avibactam based on MIC_50/90_ values (Table 2).

**Table 1:** Antimicrobial activity of ceftibuten-avibactam against the most common species and resistant subsets of Enterobacterales causing complicated urinary tract infections

Organism/organism group (no. of isolates)	No. and cumulative % of isolates inhibited at MIC (mg/L) of: ^a^												MIC_50_	MIC_90_
	≤ 0.015	0.03	0.06	0.12	0.25	0.5	1	2	4	8	16	> 16		
All Enterobacterales (3216)	1,432 44.5	1,043 77.0	460 91.3	104 94.5	51 96.1	42 97.4	32 **98.4**	24 99.1	11 99.5	3 **99.6**	2 99.6	12 100.0	0.03	0.06
ESBL-phenotype (541)^b^	120 22.2	179 55.3	146 82.3	34 88.5	26 93.3	17 96.5	6 **97.6**	2 98.0	2 98.3	0 **98.3**	1 98.5	8 100.0	0.03	0.25
MDR (317)	49 15.5	82 41.3	85 68.1	27 76.7	25 84.5	17 89.9	7 **92.1**	5 93.7	5 95.3	1 **95.6**	2 96.2	12 100.0	0.06	1
CRE (57)	3 5.3	2 8.8	10 26.3	5 35.1	12 56.1	9 71.9	1 **73.7**	1 75.4	2 78.9	0 **78.9**	0 78.9	12 100.0	0.25	> 16
Levofloxacin-NS (789)	222 28.1	256 60.6	188 84.4	50 90.7	32 94.8	18 971	6 **97.8**	3 98.2	3 98.6	0 **98.6**	1 98.7	10 100.0	0.03	0.12
Nitrofurantoin-NS (1,038)	499 48.1	243 71.5	130 84.0	49 88.7	36 92.2	24 94.5	20 **96.4**	18 98.2	7 98.8	2 **99.0**	1 99.1	9 100	0.03	0.25
TMP-SMX-NS (856)	303 35.4	318 72.5	141 89.0	35 93.1	23 95.8	13 97.3	6 **98.0**	4 98.5	3 98.8	0 **98.8**	1 98.9	9 100.0	0.03	0.12
*E. coli* (1,912)	793 41.5	741 80.2	306 96.2	43 98.5	13 99.2	6 99.5	5 **99.7**	0 99.7	1 99.8	0 **99.8**	1 99.8	3 100.0	0.03	0.06
*K. pneumoniae* (476)	200 42.0	162 76.1	59 88.4	16 91.8	19 95.8	12 98.3	1 **98.5**	1 98.7	1 98.9	0 **98.9**	0 98.9	5 100.0	0.03	0.12
*P. mirabilis* (205)	187 91.2	12 97.1	3 98.5	0 98.5	2 99.5	0 99.5	0 **99.5**	1 100.0					≤ 0.015	≤ 0.015
Indole-positive Proteeae (131)	108 82.4	12 91.6	4 94.7	4 97.7	2 99.2	0 99.2	0 **99.2**	0 99.2	0 99.2	0 **99.2**	0 99.2	1 100.0	≤ 0.015	0.03
*E. cloacae* species complex (126)	6 4.8	25 24.6	26 45.2	12 54.8	3 57.1	8 63.5	13 **73.8**	17 87.3	9 94.4	3 **96.8**	1 97.6	3 100.0	0.12	4

^a^Values in bold indicate percentages inhibited at the ceftibuten susceptible breakpoint published by EUCAST (≤ 1 mg/L) and CLSI (≤ 8 mg/L) [16, 17]

^b^*E. coli*, *K. pneumoniae*, and *P. mirabilis* isolates were categorized as exhibiting an ESBL phenotype based on CLSI criteria, i.e., the isolate had an elevated MIC value (≥ 2 mg/L) for ceftazidime, ceftriaxone, or aztreonam [CLSI 2022]; however, the mechanism responsible for decreased susceptibility to these β-lactams could be other than ESBL production, such as production of plasmidic AmpC and/or carbapenemases

Abbreviations: *CRE*, carbapenem-resistant Enterobacterales; *ESBL*, extended-spectrum β-lactamase; *NS*, nonsusceptible; *TMP-SMX*, trimethoprim-sulfamethoxazole

**Table 2:** Antimicrobial activity of ceftibuten-avibactam in comparison to oral and intravenous comparator agents tested against 3216 Enterobacterales

Antimicrobial agent	mg/L		CLSI^a^				EUCAST^a^		
	MIC_50_	MIC_90_	%S	%I	%R	%S		%I	%R
All Enterobacterales (3216)									
Ceftibuten-avibactam	0.03	0.06	[99.6]^b^			[98.4]^b^			
Ceftibuten	0.25	16	89.3^c^	2.1	8.6	79.5^c^			20.5
Cefuroxime	4	> 64	61.2^d^	13.4	25.4	71.2^c^			28.8^c^
Cefazolin	4	> 32	66.6^c^		33.4^c^				
Cephalexin	8	> 256	66.6^c^		33.4^c^	67.5^c^			32.5^c^
Levofloxacin	0.06	16	75.4	3.3	21.3	75.4		3.3	21.3
Ciprofloxacin	0.03	> 4	72.9	3.6	23.5	72.9		3.6	23.5
Nitrofurantoin	16	> 64	67.7	13.7	18.6				
TMP-SMX	≤ 0.12	> 4	73.4		26.6	73.4		0.7	26.0
Ceftazidime-avibactam	0.12	0.25	99.6		0.4	99.6			0.4
Ceftolozane-tazobactam	0.25	1	94.1	1.5	4.4	94.1			5.9
Piperacillin-tazobactam	2	16	87.7	3.2	9.1	87.7			12.3
Ceftriaxone	≤ 0.06	> 8	79.0	0.7	20.2	79.0^e^		0.7	20.2^e^
Meropenem	0.03	0.06	98.2	0.1	1.7	98.3		0.7	1.0
Gentamicin	1	4	90.6	0.6	8.9	89.8^f^			10.2^f^
Amikacin	2	4	99.1	0.4	0.4	97.8^f^			2.2^f^
Colistin	0.25	> 8		85.6	14.4	85.6			14.4
ESBL-phenotype (541)^g^									
Ceftibuten-avibactam	0.03	0.25	[98.3]^b^			[97.6]^b^			
Ceftibuten	8	> 16	56.7^b^	11.1	32.2	13.1^b^			86.9
Levofloxacin	8	32	24.6	9.3	66.1	24.6		9.3	66.1
Ciprofloxacin	> 4	> 4	17.7	8.1	74.1	17.7		8.1	74.1
Nitrofurantoin	16	> 64	63.0	10.9	26.1				
TMP-SMX	> 4	> 4	32.0		68.0	32.0		1.1	66.9
Ceftazidime-avibactam	0.12	1	98.2		1.8	98.2			1.8
Ceftolozane-tazobactam	0.5	> 16	82.2	3.7	14.1	82.2			17.8
Meropenem	0.03	0.5	91.3	0.4	8.3	91.7^e^		3.7	4.6^e^
Gentamicin	1	> 16	66.7	1.7	31.6	66.0^f^			34.0^f^
Amikacin	4	8	95.6	2.0	2.4	90.8^f^			9.2^f^
Colistin	0.25	1		90.7	9.3	90.7			9.3
MDR (317)									
Ceftibuten-avibactam	0.06	1	[95.6]^b^			[92.1]^b^			
Ceftibuten	16	> 16	41.3^c^	10.4	48.3^c^	11.7^f^			88.3
Levofloxacin	16	> 32	11.7	9.1	79.2	11.7		9.1	79.2
Ciprofloxacin	> 4	> 4	4.7	6.0	89.3	4.7		6.0	89.3^e^
Nitrofurantoin	16	> 64	46.1	16.1	37.9				
TMP-SMX	> 4	> 4	25.6		74.4	25.6		1.6	72.8
Ceftazidime-avibactam	0.25	2	95.6		4.4	95.6			4.4
Ceftolozane-tazobactam	1	> 16	63.6	6.6	29.7	63.6			36.4
Ceftriaxone	> 8	> 8	4.4	0.6	95.0	4.4^e^		0.6	95.0^e^
Meropenem	0.03	8	82.0	1.3	16.7	83.3^e^		6.9	9.8^e^
Gentamicin	> 16	> 16	41.6	2.8	55.5	39.7^f^			60.3^f^
Amikacin	4	16	91.8	3.8	4.4	81.7^f^			18.3^f^
Colistin	0.25	> 8		83.6	16.4	83.6			16.4
CRE (57)									
Ceftibuten-avibactam	0.25	> 16	[78.9]^b^			[73.7]^b^			
Ceftibuten	> 16	> 16	19.3^c^	3.5	77.2^c^	5.3^c^			94.7^c^
Levofloxacin	32	> 32	15.8	3.5	80.7	15.8		3.5	80.7
Ciprofloxacin	> 4	> 4	14.0	1.8	84.2	14.0		1.8	84.2
Nitrofurantoin	> 64	> 64	15.8	14.0	70.2				
TMP-SMX	> 4	> 4	24.6		75.4	24.6		3.5	71.9
Ceftazidime-avibactam	2	> 32	77.2		22.8	77.2			22.8
Gentamicin	1	> 16	66.7	3.5	29.8	61.4^f^			38.6^f^
Amikacin	8	> 32	71.9	14.0	14.0	61.4^f^			38.6^f^
Colistin	0.25	> 8		70.2	29.8	70.2			29.8

^a^Criteria as published by CLSI and EUCAST [16, 17]

^b^Values in brackets indicate percentage inhibited at ≤ 8 mg/L (CLSI column) and ≤ 1 mg/L (EUCAST column) for comparison to ceftibuten alone

^c^Using uncomplicated urinary tract infection only breakpoints

^d^Using oral breakpoints

^e^Using parenteral breakpoints

^f^For infections originating from the urinary tract. For systemic infections, aminoglycosides must be used in combination with other active therapy

^g^*E. coli*, *K. pneumoniae,* and *P. mirabilis* isolates were categorized as exhibiting an ESBL phenotype based on CLSI criteria, i.e., the isolate had an elevated MIC value (≥ 2 mg/L) for ceftazidime, ceftriaxone, or aztreonam [CLSI 2022]; however, the mechanism responsible for decreased susceptibility to these β-lactams could be other than ESBL production, such as production of plasmidic AmpC and/or carbapenemases

Abbreviations: *TMP-SMX*, trimethoprim-sulfamethoxazole; *CRE*, carbapenem-resistant Enterobacterales; *ESBL*, extended spectrum β-lactamase; *MDR*, multidrug-resistant

The most active oral agents after ceftibuten-avibactam (MIC_50/90_, 0.03/0.06 mg/L; 98.4% inhibited at ≤ 1 mg/L) were ceftibuten (MIC_50/90_, 0.25/16 mg/L; 89.3%/79.5% susceptible per CLSI/EUCAST), levofloxacin (MIC_50/90_, 0.06/16 mg/L; 75.4% susceptible per CLSI and EUCAST), and trimethoprim-sulfamethoxazole (TMP-SMX; MIC_50/90_, ≤ 0.12/ > 4 mg/L; 73.4% susceptible per CLSI and EUCAST; Table 2).

Ceftibuten-avibactam retained potent activity and broad coverage against ESBL-phenotype (MIC_50/90_, 0.03/0.25 mg/L; 97.6%/98.3% inhibited at ≤ 1/ ≤ 8 mg/L), MDR (MIC_50/90_, 0.06/1 mg/L; 91.6%/95.3% inhibited at ≤ 1/ ≤ 8 mg/L), and CRE isolates (MIC_50/90_, 0.25/ > 16 mg/L; 73.7%/78.9% inhibited at ≤ 1/ ≤ 8 mg/L; Table 1); all other oral agents showed limited activity against these resistant subsets (Table 2). Moreover, ceftibuten-avibactam activity against CREs was similar to ceftazidime-avibactam (MIC_50/90_, 2/ > 32 mg/L; 77.2% susceptible per CLSI and EUCAST; Table 2). Ceftibuten-avibactam was also active against isolates non-susceptible to levofloxacin (MIC_50/90_, 0.03/0.12 mg/L; 97.8%/98.6% inhibited at ≤ 1/ ≤ 8 mg/L), nitrofurantoin (MIC_50/90_, 0.03/0.25 mg/L; 96.4%/99.0% inhibited at ≤ 1/ ≤ 8 mg/L), or TMP-SMX (MIC_50/90_, 0.03/0.12 mg/L; 98.0%/98.8% inhibited at ≤ 1/ ≤ 8 mg/L), which are oral agents commonly used to treat UTI (Table 1).

The 3 Enterobacterales species most frequently isolated from UTI, *E. coli*, *K. pneumoniae*, and *P. mirabilis*, were very susceptible to ceftibuten-avibactam, with 98.5% to 99.7% of isolates inhibited at ≤ 1 mg/L (Table 1). Indole-positive Proteeae were also highly susceptible to ceftibuten-avibactam (MIC_50/90_, ≤ 0.015/0.03 mg/L; 99.2% inhibited at ≤ 1 mg/L and ≤ 8 mg/L), while *Enterobacter cloacae* species complex isolates exhibited slightly higher ceftibuten-avibactam MIC values (MIC_50/90_, 0.12/4 mg/L; 73.8%/96.8% inhibited at ≤ 1/ ≤ 8 mg/L) than those other Enterobacterales species (Table 1).

Among isolates with ceftibuten-avibactam MICs > 2 mg/L (*n* = 28; 0.9%), susceptibility to meropenem and ceftazidime-avibactam was 42.9%/50.0% (CLSI/EUCAST) and 57.1% (CLSI and EUCAST), respectively; whereas among isolates with ceftibuten-avibactam MICs > 8 mg/L (*n* = 14; 0.4%), susceptibility to meropenem and ceftazidime-avibactam was 7.1%/14.3% (CLSI/EUCAST) and 14.3% (CLSI and EUCAST), respectively.

## Discussion

Increasing antimicrobial resistance coupled with the lack of new oral antimicrobial agents for MDR organisms represents a major challenge for the treatment of complicated and uncomplicated UTI. Infections caused by ESBL-producing Enterobacterales or CRE usually require IV antimicrobial therapy with very limited options for oral step-down treatment [7, 19].

The results of the present study showed that ceftibuten-avibactam was very active against a large collection of Enterobacterales isolates causing UTI in various regions of the world. Moreover, ceftibuten-avibactam retained strong activity against isolates with an MDR and/or ESBL phenotype, as well as isolates nonsusceptible to levofloxacin, nitrofurantoin, and/or TMP-SMX. Ceftibuten-avibactam also retained activity against most CRE isolates, inhibiting 73.7% and 78.9% at ≤ 1 mg/L and ≤ 8 mg/L, respectively. Characterization of the isolates with increased ceftibuten-avibactam MIC values (≥ 2 mg/L) is ongoing. Once completed, these characterizations will provide a better understanding of the spectrum of activity for this antimicrobial combination.

The limitations of the study should be considered when interpreting the data. The absence of fosfomycin and pivmecillinam as comparator agents represents a limitation of this investigation since these oral agents are commonly used to treat UTI in some regions. Another limitation of the study is the lack of β-lactamase characterization of ESBL-phenotype and CRE isolates, as well as those with decreased susceptibility to ceftibuten-avibactam. Despite the limitations of the study, the results presented here provide valuable information on the in vitro activity of this novel agent against contemporary isolates from patients with UTI. In summary, these results indicate that ceftibuten-avibactam may represent a valuable addition for the treatment of UTI caused by MDR Enterobacterales. Further pharmacokinetic/pharmacodynamic and clinical studies are warranted to define the role of ceftibuten-avibactam for the treatment of UTI.

## Supplementary Information

Below is the link to the electronic supplementary material.

Supplementary file1 (DOCX 83 KB)

## Data Availability

The data evaluated in this investigation is part of the SENTRY Antimicrobial Surveillance Program; available at: https://sentry-mvp.jmilabs.com/app/sentry-public

## Declarations

JMI Laboratories was contracted to perform services in 2018–2021 for AbbVie, Achaogen, Inc., Affinity Biosensors, Albany College of Pharmacy and Health Sciences, Allecra Therapeutics, Allergan, Amicrobe Advanced Biomaterials, Inc., American Proficiency Institute, AmpliPhi Biosciences Corp., Amplyx Pharma, Antabio, Arietis Corp., Arixa Pharmaceuticals, Inc., Artugen Therapeutics USA, Inc., Astellas Pharma Inc., Athelas, Becton, Basilea Pharmaceutica Ltd., Bayer AG, Becton, Beth Israel Deaconess Medical Center, BIDMC, bioMerieux, Inc., bioMerieux SA, BioVersys Ag, Boston Pharmaceuticals, Bugworks Research Inc., CEM-102 Pharmaceuticals, Cepheid, Cidara Therapeutics, Inc., Cipla, Contrafect, Cormedix Inc., Crestone, Inc., Curza, CXC7, DePuy Synthes, Destiny Pharma, Dickinson and Company, Discuva Ltd., Dr. Falk Pharma GmbH, Emery Pharma, Entasis Therapeutics, Eurofarma Laboratorios SA, Fedora Pharmaceutical, F. Hoffmann-La Roche Ltd., Fimbrion Therapeutics, US Food and Drug Administration, Fox Chase Chemical Diversity Center, Inc., Gateway Pharmaceutical LLC, GenePOC Inc., Geom Therapeutics, Inc., GlaxoSmithKline plc, Guardian Therapeutics, Hardy Diagnostics, Harvard University, Helperby, HiMedia Laboratories, ICON plc, Idorsia Pharmaceuticals Ltd., IHMA, Iterum Therapeutics plc, Janssen Research & Development, Johnson & Johnson, Kaleido Biosciences, KBP Biosciences, Laboratory Specialists, Inc., Luminex, Matrivax, Mayo Clinic, Medpace, Meiji Seika Pharma Co., Ltd., Melinta Therapeutics, Inc., Menarini, Merck & Co., Inc., Meridian Bioscience Inc., Micromyx, Microchem Laboratory, MicuRx Pharmaceutics, Inc., Mutabilis Co., N8 Medical, Nabriva Therapeutics plc, National Institutes of Health, NAEJA-RGM, National University of Singapore, North Bristol NHS Trust, Novartis AG, Novome Biotechnologies, Oxoid Ltd., Paratek Pharmaceuticals, Inc., Pharmaceutical Product Development, LLC, Polyphor Ltd., Prokaryotics Inc., QPEX Biopharma, Inc., Ra Pharmaceuticals, Inc., Rhode Island Hospital, RIHML, Roche, Roivant Sciences, Ltd., Safeguard Biosystems, Salvat, Scynexis, Inc., SeLux Diagnostics, Inc., Shionogi and Co., Ltd., SinSa Labs, Specific Diagnostics, Spero Therapeutics, Summit Pharmaceuticals International Corp., SuperTrans Medical LT, Synlogic, T2 Biosystems, Taisho Pharmaceutical Co., Ltd., TenNor Therapeutics Ltd., Tetraphase Pharmaceuticals, The Medicines Company, The University of Queensland, Theravance Biopharma, Thermo Fisher Scientific, Tufts Medical Center, Universite de Sherbrooke, University of Colorado, University of Southern California-San Diego, University of Iowa, University of Iowa Hospitals and Clinics, University of North Texas Health Science Center, University of Wisconsin, UNT System College of Pharmacy, URMC, UT Southwestern, VenatoRx, Viosera Therapeutics, Vyome Therapeutics Inc., Wayne State University, Wockhardt, Yukon Pharmaceuticals, Inc., Zai Lab, and Zavante Therapeutics, Inc. There are no speakers’ bureaus or stock options to declare.
