# Supplementary material for: Antimicrobial activity of ceftibuten-avibactam against a global collection of Enterobacterales from patients with urinary tract infections (2021)
Source: Eur J Clin Microbiol Infect Dis. 2023 Feb 22;42(4):453–9. doi: 10.1007/s10096-023-04562-4 (PMC9998307; doi:10.1007/s10096-023-04562-4)

**Supplemental Figure S1.** Frequency of Enterobacterales isolated from patients with urinary tract infections in the United Sates (A) and Europe (B)

#
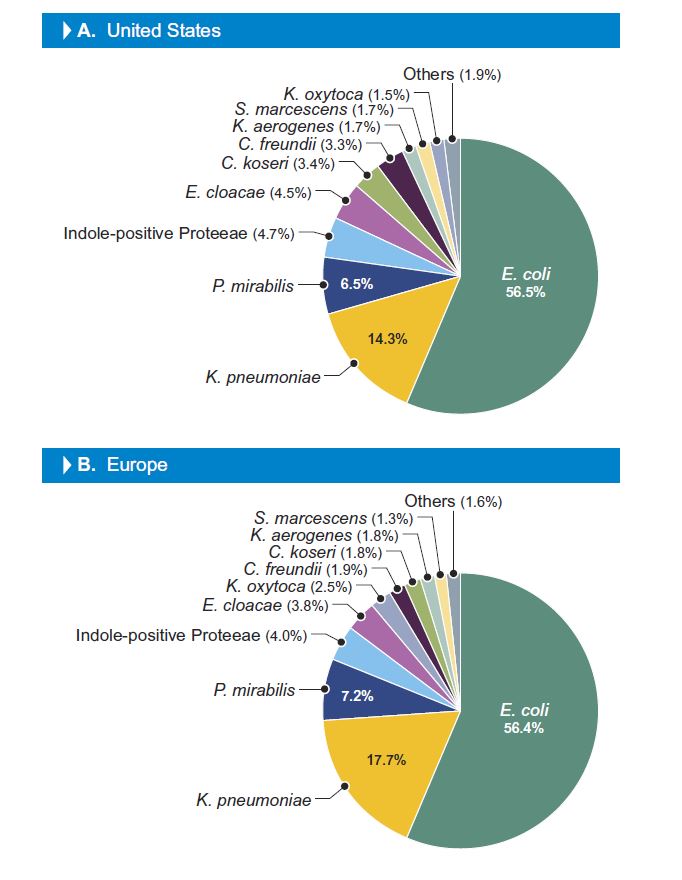

Supplement: Supplementary file 1 — Supplementary file1 (DOCX 83 KB) [file 10096_2023_4562_MOESM1_ESM.docx]
